# Thrombin Contributes to Anti-myeloperoxidase Antibody Positive IgG-Mediated Glomerular Endothelial Cells Activation Through SphK1-S1P-S1PR3 Signaling

**DOI:** 10.3389/fimmu.2019.00237

**Published:** 2019-02-15

**Authors:** Xiao-Jing Sun, Min Chen, Ming-Hui Zhao

**Affiliations:** ^1^Renal Division, Department of Medicine, Peking University, First Hospital, Peking University Institute of Nephrology, Beijing, China; ^2^Key Laboratory of Renal Disease, Ministry of Health of China, Beijing, China; ^3^Key Laboratory of Chronic Kidney Disease Prevention and Treatment, Ministry of Education, Peking-Tsinghua Center for Life Sciences, Peking University, Beijing, China

**Keywords:** ANCA, vasculitis, thrombin, sphingosine-1-phosphate, endothelium

## Abstract

**Background:** Activation of coagulation system plays an important role in antineutrophil cytoplasmic antibody (ANCA)-associated vasculitis (AAV) pathogenesis. Thrombin, generated during coagulation could disrupt endothelial barrier integrity through protease-activated receptor 1 (PAR1). Our previous study found that sphingosine-1-phosphate (S1P) contributed to myeloperoxidase (MPO)-ANCA-positive IgG-induced glomerular endothelial cell (GEnC) activation through a S1P receptor (S1PR)-dependent route. In recent years, S1P signaling was reported to be involved in thrombin effects on endothelial cells. This current study investigated whether the interaction between thrombin-PAR and S1P-S1PR signaling contributed to MPO-ANCA-positive IgG-induced GEnC dysfunction.

**Methods:** The effect of thrombin on GEnC activation was analyzed from three aspects. First, morphological alteration of GEnCs was observed. Second, permeability assay was performed to determine GEnC monolayer activation quantitatively. Third, endothelin-1 (ET-1) levels were measured. Expression levels of sphingosine kinases (SphKs) and S1PRs were detected. In addition, antagonists of PAR1 and S1PR3 were employed to determine their roles. Eventually, PAR1 and tissue factor (TF) expression levels as well as TF procoagulant activity were analyzed.

**Results:** Thrombin induced further damage of tight junction, increase in endothelial monolayer permeability as well as upregulation of ET-1 levels in GEnCs stimulated with MPO-ANCA-positive IgG. Blocking PAR1 downregulated ET-1 levels in the supernatants of GEnCs treated by thrombin plus MPO-ANCA-positive IgG. Expression levels of SphK1, S1PR3 increased significantly in GEnCs treated with thrombin plus MPO-ANCA-positive IgG. S1P upregulated PAR1 and TF expression, and enhanced procoagulant activity of TF in MPO-ANCA-positive IgG-stimulated GEnCs.

**Conclusion:** Thrombin synergized with SphK1-S1P-S1PR3 signaling pathway to enhance MPO-ANCA-positive IgG-mediated GEnC activation.

## Introduction

Anti-neutrophil cytoplasmic antibody (ANCA)-associated vasculitis (AAV) consists of eosinophilic granulomatosis with polyangiitis (EGPA), granulomatosis with polyangiitis (GPA) and microscopic polyangiitis (MPA) (1). AAV is characterized by necrotizing inflammation of the small blood vessels, which involves glomerular endothelial cell (GEnC) injury in particular. The serological hallmarks for AAV are ANCAs against either proteinase 3 (PR3) or myeloperoxidase (MPO) (2, 3). The majority of Chinese AAV patients are MPO-ANCA-positive, as reported in our previous studies (4, 5). In addition, cumulating evidences suggest that MPO-ANCAs cause GEnC activation and injury directly in AAV (6, 7).

Sphingosine-1-phosphate (S1P) is a bioactive sphingolipid metabolite and produced by phosphorylation of sphingosine by sphingosine kinases (SphKs). S1P is the ligand for five G-protein-coupled receptors (GPCRs) named S1PR1-5 (8). S1P and S1PRs participate in the pathogenesis of a variety of vascular inflammatory conditions including ischemia-reperfusion injury, atherosclerosis and sepsis (9–11). In recent years, clinical trials that targeted S1PRs for autoimmune diseases have attracted wide interest. Of note, FTY720 (Fingolimod, Gilenya, Novartis), a functional antagonist of S1PR1, 3, 4, and 5, has already been approved and used in treating multiple sclerosis (12–14). Moreover, cumulating evidences supported a vital role of FTY720 in endothelial barrier enhancement both *in vivo* and *in vitro* (15–17). In our previous studies, we found that the circulating levels of S1P and the renal expression of S1PRs correlated with renal involvement and disease activity of AAV. In addition, it was found that S1P enhanced MPO-ANCA-positive IgG-induced GEnC activation through S1PR2-5 and RhoA signaling pathway (18–20). All these studies indicated a pathogenic role of S1P in AAV.

Although the pathogenesis of AAV is not yet fully clear, the interaction among ANCA, neutrophils and complement activation is of vital importance in the development of this disease [reviewed by Chen et al. (21)]. In recent years, more and more evidence has suggested that activation of coagulation system may also play an important role. Patients with AAV are in a hypercoagulable state, with an increased risk of developing venous thromboembolic events (22, 23). Moreover, the interaction between coagulation and complement system also contributes to the pathogenesis of glomerular capillary tuft infarction and to the increased frequency of thromboembolic events in AAV. Some serine proteases from the coagulation cascade, in particular plasmin and thrombin, can directly activate C3 and C5, independent of the traditional C3/C5 convertase (24, 25). C5a-primed neutrophils produce tissue-factor-expressing microparticles and neutrophil extracellular traps (NETs) after stimulation with ANCAs, which subsequently activate the coagulation system (26). Platelets are activated *via* thrombin-PARs pathway and can activate the alternative complement pathway in AAV (27).

The coagulation system is initiated in two distinct mechanisms: the contact pathway and the tissue factor (TF) pathway. Both pathways result in the generation of thrombin, the best-characterized activator of protease-activated receptors (PARs) (28). PARs are a family of G protein-coupled receptors including 4 members named PAR1-4. PAR1 is the major effector of thrombin signaling in most cell types including endothelial cells. Thrombin activates PAR1 by catalyzing the cleavage of the Arg41-Ser42 peptide bond on the N-terminal extracellular domain of the receptor (29). It was reported that thrombin-activated PAR1 could induce disruption of endothelial barrier integrity (30).

Thrombin effects in endothelial cells involve S1P signaling. According to Tauseef et al. SphK1-S1P-S1PR1 signaling could counteract the detrimental effect of thrombin-PAR1 signaling on endothelial barrier function. On the one hand, thrombin-activated-PAR1 interrupts endothelial barrier integrity *via* Rho signaling pathway; on the other hand, thrombin also induces expression of SphK1 and increases S1P generation, which in turn transactivates S1PR1 leading to the activation of Rac1 signaling pathway. This effect improves endothelial integrity to counteract and limit thrombin-induced endothelial damage and vascular leakage (31). However, some other studies revealed a synergistic effect of S1P on thrombin-induced endothelial dysfunction, including enhanced NF-κB binding activity and TF expression in endothelial cells (32, 33). Given the potential effect of thrombin-PAR and SphK-S1P-S1PR signaling on regulating endothelial barrier function, our current study aimed to investigate whether the interaction between thrombin-PAR and SphK-S1P-S1PR signaling participated in MPO-ANCA-positive IgG-induced GEnC dysfunction.

## Materials and Methods

### Cell Culture

Primary human glomerular endothelial cells (GEnC; ScienCell, San Diego, CA, USA) were cultured in endothelial cell basal medium (ECM) (ScienCell San Diego, CA, USA) supplemented with 10% fetal bovine serum (FBS), 1% penicillin/streptomycin and 1% endothelial cell growth factor. Cultures were grown in an atmosphere of 5% CO_2_ at 37°C. After starving in ECM with additional 0.5% FBS for 8 h, GEnC in selected wells were washed with phosphate buffered saline (PBS) and then stimulated with thrombin (Sigma, Darmstadt, Germany), MPO-ANCA-positive IgG, normal IgG or 2 μmol/L S1P (Sigma, Darmstadt, Germany), which was comparable to the levels of circulating S1P in AAV patients at active stage, as demonstrated by our previous study (18).

### Preparation of Immunoglobulin (Ig)Gs

Preparation of IgGs was performed according to the methods described previously (34). MPO-ANCA-positive IgGs and normal IgGs and were prepared from plasma exchange liquid of eight patients with active MPO-ANCA-positive primary small vessel vasculitis and plasma of six healthy donors, respectively. Then we further screened the prepared IgGs for the presence of anti-endothelial cell antibody (AECA) through an ELISA method described previously (35), and AECA-positive IgGs were excluded in our following experiments. Eventually, normal IgGs from plasma of five healthy donors and MPO-ANCA-positive IgGs from plasma exchange liquid of five AAV patients were included, respectively. Our research was in compliance with the Declaration of Helsinki and approved by the clinical research ethics committee of the Peking University First Hospital.

### Measurements of GEnC Activation and Injury

#### Immunofluorescence Staining of Zonula Occludens-1 (ZO-1) and Vascular Endothelial (VE)-Cadherin

As important markers for endothelial barrier function, the distribution of the tight junction scaffolding protein ZO-1 and adherens junction protein VE-cadherin were observed (36). After relevant treatment, GEnCs were washed in PBS and fixed with 4% formaldehyde for 30 min. Next, the GEnCs were permeabilized with 0.5% Triton X-100, washed and blocked with 5% BSA for 1 h at room temperature. After incubation with primary antibodies (ZO-1, dilution 1/100, Life, Carlsbad, CA, USA; VE-cadherin, dilution 1/200, Abcam, Cambridge, MA, USA) at 4°C overnight and a thorough wash in PBS, the GEnCs were incubated with fluorescein isothiocyanate (FITC)-conjugated secondary antibodies (for the detection of ZO-1, dilution 1/200, Jackson ImmunoResearch, West Grove, PA, USA; for the detection of VE-cadherin, dilution 1/500, Abcam, Cambridge, MA, USA) at 37°C for 1 h. Eventually, the specimens were stained with 10 μg/ml 4',6-diamidino-2-phenylindole (DAPI) and mounted with Mowiol. The immunofluorescence staining was photographed by a fluorescence microscope (Nikon Eclipse 90i, Nikon Instruments Inc., Tokyo, Japan). At least 10 visual fields per slide of GEnCs at ×400 were observed blindly. Image J software (National Institutes of Health, Bethesda, MD, USA) was used to evaluate the immunofluorescence staining of ZO-1 and VE-cadherin. Positive signals were quantified as signal intensity.

#### Permeability Assay

The permeability of GEnC monolayers was determined using Costar Transwell plate with 0.5-μm porous filters and FITC-labeled BSA (Sigma-Aldrich, Darmstadt, Hessen, Germany), as described previously (37). GEnCs were grown on the upper chamber of Costar Transwell until confluent. The tracer protein FITC-albumin was added to the upper chamber after relevant stimulation. After incubation at 37°C for 30 min, samples from both the upper and lower chambers were collected for fluorometric analysis. Fluorescent intensity (FI) was measured using a microplate fluorescence reader (Tristar^TM^ LB941, Berthold, Germany) with filter settings of 485 nm (excitation) and 538 nm (emission). Eventually, these fluorescence readings were used for calculation of the permeability coefficient, which is indicative of vascular barrier disruption. The permeability coefficient was calculated according to the following formula:

Permeability coefficient = FI (lower chamber) × 100% / (FI (upper chamber) + FI (lower chamber)).

#### Evaluation of Endothelium Activation by Endothelin-1 (ET-1) Quantification

As a biomarker of endothelial cell activation and injury (38), ET-1 levels in GEnC supernatants were measured using commercial ELISA kits (R&D, Minneapolis, MN, USA).

### Measurement of SphKs, S1PRs, PARs, and TF

SphK1 and 2, S1PR1-5, PAR1, and TF expression levels were determined by quantitative real-time polymerase chain reaction (qRT-PCR). GEnCs were washed in Dulbecco's phosphate-buffered saline (D-PBS) and total RNA was extracted using a commercial RNA purification kit (Thermo scientific, Waltham, MA, USA). Concentration and purity of RNA samples were determined by reading absorbance at 260 and 280 nm with a spectrophotometer (Nanodrop, Thermo fisher scientific, Wilmington, DE, USA). After cDNA synthesis using GoScript™Reverse Transcriptase (Promega, Madison, WI, USA), mRNA levels were determined by quantitative polymerase chain reaction (q-PCR) on an Applied Biosystems system (ViiA7) using Power SYBR® Green PCR Master Mix (Applied Biosystems, Austin, TX, USA). Amplifications were pre-incubation at 95°C for 10 min, followed by 40 cycles of 94°C for 30 s, 60°C for 30 s and 72°C for 30 s. Values were expressed as 2^−ΔΔ*CT*^. β-actin and GAPDH were used as endogenous controls. Primers used are listed in Table 1.

**Table 1:** Sequences of PCR primers used.

**Gene**	**Forward primer 5'-3'**	**Reverse primer 5'-3'**
SphK1	AAACCCCTGTGTAGCCTCCC	AGCAGGTTCATGGGT GACAG
SphK2	GCACAGCAACAGTGAGCA-3'	GAGCCTGAG TGAGTG GGA
S1PR1	CACTCTGACCAACAAGGAGATG	GATGATGGGTCGCTTGAATTTG
S1PR2	AAGTTCCACTCGGCAATGTA	AGCCAGAGAGCAAGGTATTG
S1PR3	TCTCCGAAGGTCAAGGAAGA	TCAGTTGCAGAAGATCCCATTC
S1PR4	CTGAAGACGGTGCTGATGAT	CAGAGGTTGGAGCCAAAGA
S1PR5	GGTCATCGTCCTGCATTACA	CTAGATTCTCTAGCACGATGA AGG
PAR1	CAGGCACTACAAATACTGTGG	TGTAGACTTGATTGACGGGTT
TF	GCCAGGAGAAAGGGGAAT	CAGTGCAATATAGCATTTGCA GTAGC
β-actin	GGACCTGACTGACTACCTCAT	CGTAGCACAGCTTCTCCTTAAT
GAPDH	GAGTCAACGGATTTGGTCGT	GACAAGCTTCCCGTTCTCAG

### Detection of SphK1 by Western Blot

Samples were incubated for 10 min at 95°C in loading buffer. Samples were then subjected to electrophoresis on 10% SDS-polyacrylamide gels and transferred to nitrocellulose membranes. The membranes were incubated with primary antibodies (for the detection of SphK1, dilution 1/1000, Abcam, Cambridge, MA, USA; for the detection of β-actin, dilution 1/1000, Santa Cruz, Dallas, TX, USA) followed by horseradish peroxidase-conjugated secondary antibodies (each diluted 1:2000; both from Proteintech, Chicago, IL, USA). Proteins were visualized on autoradiographic film using an ECL Plus Western blot detection system (GE Healthcare).

### Inhibition of PAR1 and S1PR3

RWJ 56110 (RWJ; Tocris, Louis, MO, USA) is a selective PAR1 antagonist (39). TY52156 (TY; Tocris, Louis, MO, USA) is a specific antagonist for S1PR3 (40). In thrombin-induced ET-1 expression assay, GEnCs were incubated with RWJ and TY for different doses and time points. Eventually, 1 μM RWJ at 15 min and 1 μM TY at 15 min were selected for the experiments due to the highest inhibition rates.

### TF Procoagulant Activity Assay

To analyze TF procoagulant activity, a Cell Tissue Factor Assay Kit (Genmed Scientifics Inc, Wilmington, DE, USA) was used following manufacturer's instructions. GEnCs were lysed and 50 μg proteins and ~2 × 10^6^ cells of each sample were used. Samples were incubated with prothrombin complex (including Factor II, VII, IX, X) and CaCl_2_. Reaction was terminated by adding EDTA buffer. Eventually, we added a chromogenic substrate (Spectrozyme factor Xa) and measured the absorbance at 405 nm.

### Statistical Analysis

SPSS version 13.0 (SPSS Inc., Chicago, IL, USA) was used to perform data analysis. Normality of the data was evaluated by kurtosis and skewness (both the absolute values were <3). Data was generally presented as mean ± standard deviation (SD) and compared by ANOVA followed by Bonferroni correction for multiple testing. *P* < 0.05 were considered statistically significant.

## Results

### Thrombin Amplifies MPO-ANCA-Positive IgG-Mediated GEnC Dysfunction *via* PAR1

#### Thrombin Induces GEnC Morphological Alteration of GEnC Monolayers

**Figure 1:**
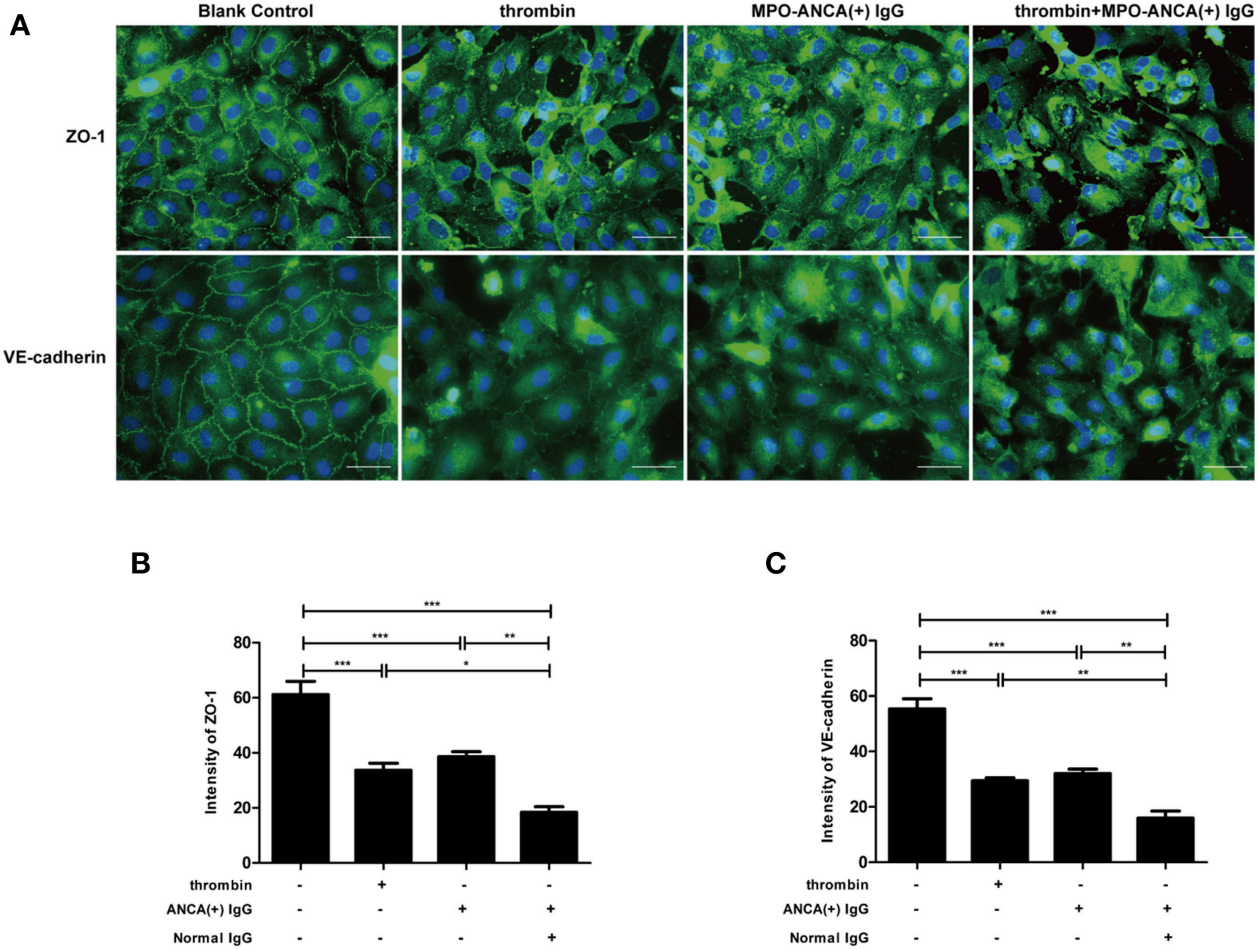
Thrombin could enhance MPO-ANCA-positive IgG-mediated GEnC activation. **(A)** Thrombin could induce alterations in cellular morphology of GEnCs in the presence of MPO-ANCA-positive IgG. **(B)** Quantitive assessment of ZO-1 in GEnCs upon stimulation by thrombin and MPO-ANCA-positive IgG. **(C)** Quantitive assessment of VE-cadherin in GEnCs upon stimulation by thrombin and MPO-ANCA-positive IgG. Bars represent mean ± SD of repeated measurements of five independent experiments or donors. *P < 0.05, **P < 0.01, ***P < 0.001.

Immunofluorescence staining of ZO-1 and VE-cadherin were performed to observe the structure of the tight junction and adherens junction in GEnCs, respectively. We found that compared with untreated cells, the application of thrombin or MPO-ANCA-positive IgG alone could disrupt tight junction and adherens junction structures (33.71 ± 5.65 vs. 61.14 ± 10.83, *P* < 0.001; 38.60 ± 4.05 vs. 61.14 ± 10.83, *P* < 0.001; 32.04 ± 3.63 vs. 55.39 ± 8.11, *P* < 0.001; 29.44 ± 2.41 vs. 55.39 ± 8.11, *P* < 0.001, respectively). Moreover, combined application of thrombin and MPO-ANCA-positive IgG induced further damage of tight junction and adherens junction compared with all the above-mentioned cell groups (18.43 ± 4.46 vs. 61.14 ± 10.83, *P* < 0.001; 18.43 ± 4.46 vs. 33.71 ± 5.65, *P* < 0.05; 18.43 ± 4.46 vs. 38.60 ± 4.05, *P* < 0.01; 15.98 ± 5.57 vs. 55.39 ± 8.11, *P* < 0.001; 15.98 ± 5.57 vs. 32.04 ± 3.63, *P* < 0.01; 15.98 ± 5.57 vs. 29.44 ± 2.41, *P* < 0.01, respectively) (Figure 1). These data revealed that thrombin synergized with MPO-ANCA-positive IgG to exert damage effects on endothelial barrier integrity.

#### Thrombin Induces Increased Endothelial Permeability in GEnC Monolayers

**Figure 2:**
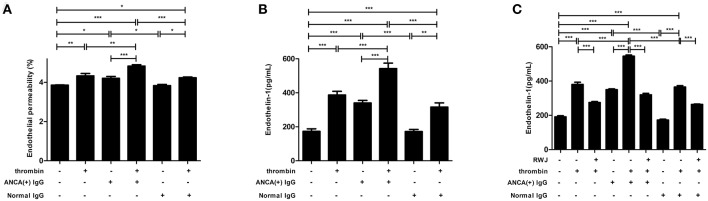
**(A)** Thrombin could induce increased endothelial permeability of GEnC monolayers in the presence of MPO-ANCA-positive IgG. **(B)** Thrombin could upregulate ET-1 levels in the supernatant of GEnCs in the presence of MPO-ANCA-positive IgG. **(C)** PAR1 mediated the thrombin-induced ET-1 upregulation in GEnCs in the presence of MPO-ANCA-positive IgG. Bars represent mean ± SD of repeated measurements of five independent experiments or donors. *P < 0.05, **P < 0.01, ***P < 0.001.

We used a transwell system and a FITC-labeled BSA to investigate the effect of S1P on monolayer permeability in GEnCs. The results revealed that compared with untreated cells, monolayer permeability increased in GEnCs stimulated with thrombin or MPO-ANCA positive IgG alone (4.33 ± 0.27% vs. 3.86 ± 0.03%, *P* < 0.01; 4.21 ± 0.21% vs. 3.86 ± 0.03%, *P* < 0.05, respectively). Furthermore, compared with the above cells, monolayer permeability still increased significantly in GEnCs stimulated by thrombin plus MPO-ANCA-positive IgG (4.83 ± 0.15% vs. 3.86 ± 0.03%, *P* < 0.001; 4.83 ± 0.15% vs. 4.33 ± 0.27%, *P* < 0.01; 4.83 ± 0.15% vs. 4.21 ± 0.21%, *P* < 0.001, respectively) (Figure 2A). These data suggested that thrombin enhanced MPO-ANCA-positive IgG-mediated increasing of GEnC permeability.

#### Thrombin Increases ET-1 Levels in GEnC Supernatants

As a biomarker of endothelial cell activation and injury, ET-1 levels in the supernatants of GEnCs were measured. It was found that compared with unstimulated cells, cells stimulated by thrombin or MPO-ANCA-positive IgG alone, the levels of ET-1 increased significantly in GEnCs treated with thrombin and MPO-ANCA-positive IgG (542.82 ± 71.58 pg/ml vs. 173.10 ± 33.48 pg/ml, *P* < 0.001; 542.82 ± 71.58 pg/ml vs. 387.33 ± 47.89 pg/ml, *P* < 0.001; 542.82 ± 71.58 pg/ml vs. 340.47 ± 32.77 pg/ml, *P* < 0.001, respectively) (Figure 2B). Collectively, these data illustrated that thrombin synergized with MPO-ANCA-positive IgG to upregulate the levels of ET-1 in the GEnC supernatants.

#### PAR1 Mediates the Thrombin-Induced Endothelial Dysfunction

GEnCs were pre-incubated with PAR1 antagonist RWJ for 15 min before stimulation with thrombin and MPO-ANCA-positive IgG, and the ET-1 levels in the supernatants were measured. We found that the ET-1 levels reduced from 545.39 ± 15.06 pg/ml in the supernatants of GEnCs stimulated by thrombin and MPO-ANCA-positive IgG to 319.86 ± 19.07 pg/ml, upon pre-incubation with PAR1 antagonist RWJ (compared with that without the antagonist, *P* < 0.001, with the inhibition rate of 41.35 ± 3.50%) (Figure 2C). These data revealed that PAR1 mediated ET-1 upregulation in thrombin and MPO-ANCA-positive IgG-treated GEnCs.

### Thrombin Amplifies MPO-ANCA-Positive IgG-Mediated GEnC Dysfunction Through SphK-S1P-S1PR Signaling Crosstalk

#### SphK1 and S1PR3 Expression Levels Are Elevated in MPO-ANCA-Positive IgG-Treated GEnCs Upon Thrombin Stimulation

**Figure 3:**
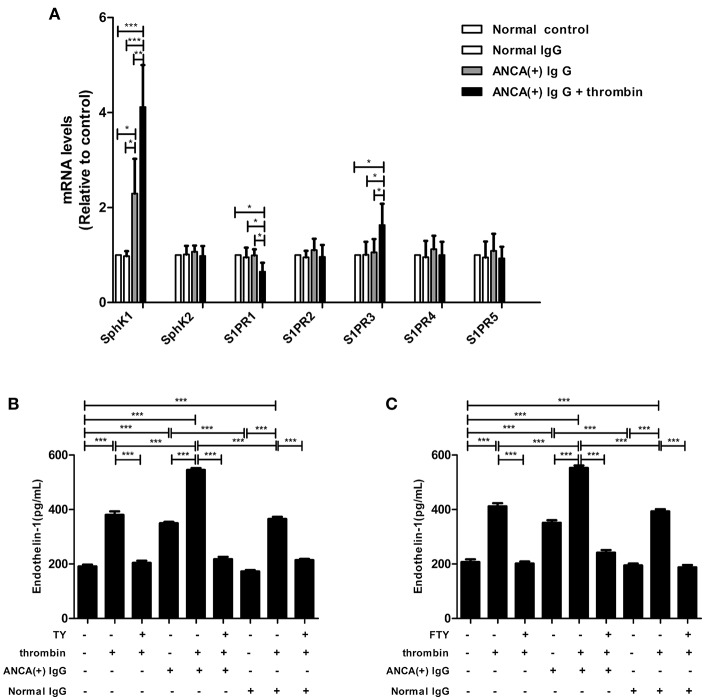
SphK1–S1P-S1PR3 signaling was involved in thrombin -induced MPO-ANCA-positive IgG-mediated GEnC activation. **(A)** SphK1 and S1PR3 expression levels were elevated in MPO-ANCA-positive IgG-treated GEnCs upon thrombin stimulation. **(B)** S1PR3 mediated the thrombin-induced ET-1 upregulation in MPO-ANCA-positive IgG-treated GEnCs. **(C)** FTY720 significantly downregulated ET-1 levels in the supernatants of GEnCs stimulated by thrombin and MPO-ANCA-positive IgG. Bars represent mean ± SD of repeated measurements of five independent experiments or donors. *P < 0.05, **P < 0.01, ***P < 0.001.

SphK1,2 and S1PR1-5 expression levels in GEnCs were measured by qRT-PCR. It was found that compared with GEnCs stimulated by MPO-ANCA-positive IgG alone, the expression levels of SphK1 and S1PR3 in GEnCs treated with thrombin plus MPO-ANCA-positive IgG increased significantly (4.12 ± 0.88 vs. 2.30 ± 0.73, *P* < 0.01; 1.63 ± 0.45 vs. 1.06 ± 0.28, *P* < 0.05, respectively), whereas S1PR1 expression level decreased significantly in GEnCs treated with thrombin plus MPO-ANCA-positive IgG (0.65 ± 0.19 vs. 0.99 ± 0.13, *P* < 0.05) (Figure 3A). The protein expression levels of SphK1 were also detected with Western blot. Consistent with the results of PCR, the expression levels of SphK1 in GEnCs stimulated with thrombin plus MPO-ANCA-positive IgG were higher than those in the other groups (Figure S1).

#### S1PR3 Mediates the Thrombin-Induced Endothelial Dysfunction

GEnCs were pre-incubated with S1PR3 antagonist TY for 15 min before stimulation with thrombin and MPO-ANCA-positive IgG, and the ET-1 levels in the supernatants were measured. We found that pre-incubation of GEnCs with TY significantly decreased ET-1 level in the supernatants of GEnCs stimulated by thrombin and MPO-ANCA-positive IgG (545.39 ± 15.06 pg/ml vs. 217.52 ± 18.99 pg/ml, *P* < 0.001, with the inhibition rate of 60.12 ± 3.48%) (Figure 3B). These data revealed that S1PR3 activation was involved in thrombin-induced ET-1 upregulation in GEnCs in the presence of MPO-ANCA-positive IgG.

We also pre-incubated GEnCs with FTY720 before stimulated with thrombin and MPO-ANCA-positive IgG, and the ET-1 levels in the supernatants were measured. We found that the ET-1 levels reduced from 552.69 ± 20.46 pg/ml in the supernatants of GEnCs stimulated by thrombin and MPO-ANCA-positive IgG to 241.53 ± 21.22pg/ml, upon pre-incubation with FTY720 (compared with those without FTY720, *P* < 0.001, with the inhibition rate of 43.70 ± 3.84%) (Figure 3C).

#### PAR1 Expression Levels Are Elevated in GEnCs Upon Stimulation by S1P

**Figure 4:**
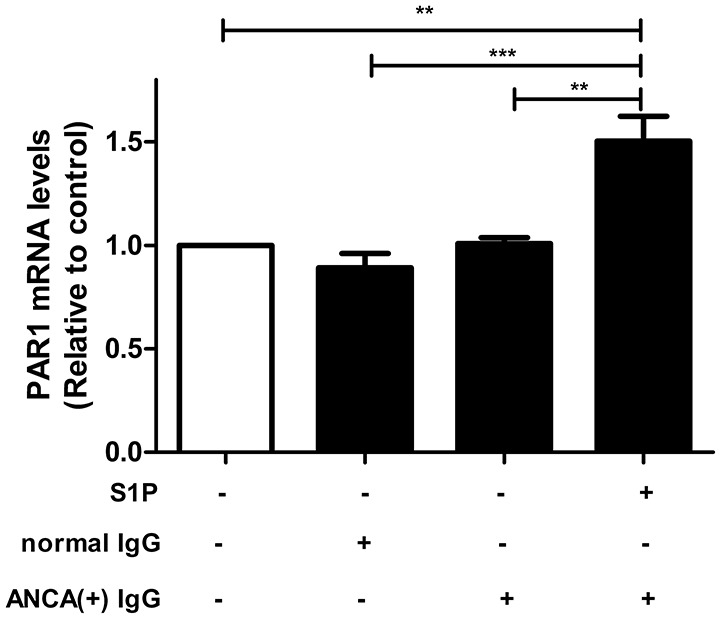
PAR1 expression levels were elevated in MPO-ANCA-positive IgG-treated GEnCs upon stimulation by S1P. Bars represent mean ± SD of repeated measurements of five independent experiments or donors. **P < 0.01, ***P < 0.001.

GEnCs were stimulated with MPO-ANCA-positive IgG plus 2 μmol/L S1P, which was comparable to the levels of circulating S1P in AAV patients at active stage, as demonstrated by our previous study (12), and PAR1 expression levels in GEnCs were measured by qRT-PCR. It was found that compared with GEnCs stimulated by MPO-ANCA-positive IgG alone, the expression levels of PAR1 in GEnCs treated with S1P plus MPO-ANCA-positive IgG increased significantly (1.50 ± 0.27 vs. 1.01 ± 0.06, *P* < 0.01) (Figure 4).

### S1P Enhances the Expression and Activity of TF in GEnCs in the Presence of MPO-ANCA-Positive IgG

**Figure 5:**
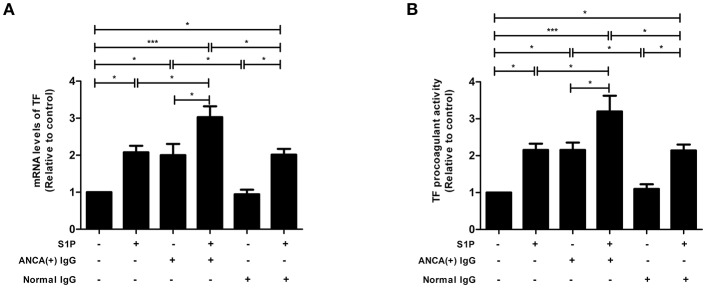
S1P enhanced the expression and activity of TF in GEnCs in the presence of MPO-ANCA-positive IgG. **(A)** S1P enhanced the expression levels of TF in GEnCs in the presence of MPO-ANCA-positive IgG. **(B)** S1P enhanced the procoagulant activity of TF in GEnCs in the presence of MPO-ANCA-positive IgG. Bars represent mean ± SD of repeated measurements of five independent experiments or donors. *P < 0.05, ***P < 0.001.

TF expression levels were detected using qRT-PCR, and it was found that compared with untreated cells, cells stimulated by S1P or MPO-ANCA-positive IgG alone, the TF levels increased significantly in GEnCs stimulated by S1P and MPO-ANCA-positive IgG (3.03 ± 0.66 vs. 1.00, *P* < 0.001; 3.03 ± 0.66 vs. 2.08 ± 0.39, *P* < 0.05; 3.03 ± 0.66 vs. 2.00 ± 0.68, *P* < 0.05, respectively) (Figure 5A). TF procoagulant activity was also measured using a commercial kit. The results demonstrated that compared with untreated cells, cells stimulated by S1P or MPO-ANCA-positive IgG alone, the activity of TF increased significantly in GEnCs stimulated by S1P and MPO-ANCA-positive IgG (3.20 ± 0.95 vs. 1.00, *P* < 0.001; 3.20 ± 0.95 vs. 2.16 ± 0.38, *P* < 0.05; 3.20 ± 0.95 vs. 2.15 ± 0.45, *P* < 0.05, respectively) (Figure 5B). Collectively, these data illustrated that S1P, with pathophysiological concentration of active AAV patients, synergized with MPO-ANCA-positive IgG to promote both the expression and activity of TF in GEnCs.

## Discussion

**Figure 6:**
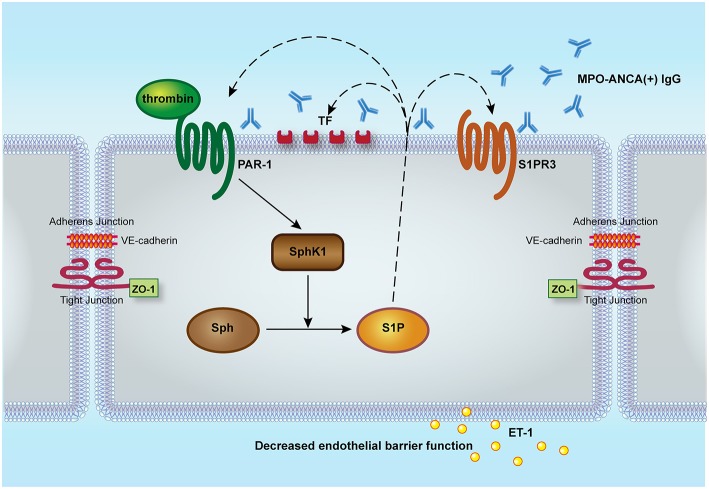
Proposed working model for the role of SphK1-S1P-S1PR3 in thrombin-induced GEnC activation in the presence of MPO-ANCA-positive IgG. Thrombin could enhance MPO-ANCA-positive IgG-induced GEnC activation and injury *via* PAR1. At the same time, thrombin might activate SphK1-S1P-S1PR3 axis in GEnCs in the presence of MPO-ANCA-positive IgG. Furthermore, S1P of pathophysiological concentration in active AAV patients might induce PAR1 expression as well as enhance both expression level and activity of tissue factor in MPO-ANCA-positive IgG-treated endothelial cells, which might further activate the coagulation system, thus forming a vicious loop. S1P, sphingosine-1-phosphate; S1PR, sphingosine-1-phosphate receptor; Sph, sphingosine; SphK, sphingosine kinases; PAR, protease-activated receptor; ET-1, endothelin-1; ZO-1, zonula occludens-1.

In our current study, we demonstrated that thrombin could enhance MPO-ANCA-positive IgG-induced GEnC activation *via* PAR1, and thrombin could activate SphK1-S1P-S1PR3 axis in GEnCs in the presence of MPO-ANCA-positive IgG. At the same time, S1P, at pathophysiological concentration in active AAV patients, could induce PAR1 expression as well as enhance both expression level and procoagulant activity of TF in MPO-ANCA-positive IgG-treated GEnCs, which may further activate the coagulation system, thus forming a vicious loop (Figure 6).

Anti-MPO antibody could cause activation of GEnCs by recognizing moesin even though MPO is not expressed in endothelial cells (41). Moesin, whose full name is membrane-organizing extension spike protein, shares certain similar sequences with those on the N-terminal region of the MPO heavy chain (7). Binding of anti-MPO antibody to moesin was able to increase permeability and to up-regulate adhesion molecules of human GEnCs (42). Recently, it was reported that thrombin was able to induce phosphorylation of moesin within seconds (43). Likewise, S1P could also cause acute and potent moesin activation (44, 45). Therefore, we speculate that moesin recognized by MPO-ANCA could be further activated by thrombin or S1P, which might cause enhanced GEnC activation *in vitro*. However, anti-PR3 antibody might induce endothelial cells dysfunction through different mechanisms. According to the study by Le Roux S et al., anti-PR3 antibodies could induce a potent inhibitor of vascular endothelial growth factor named soluble Flt1 to release from monocytes rather than endothelial cells, therefore leading to an anti-angiogenic state that hinders endothelial repair in AAV (46).

In our current study, we found that thrombin could activate SphK1-S1P-S1PR3 axis, thrombin induced upregulation of SphK1 expression levels in GEnCs in the presence of MPO-ANCA-positive IgG (confirmed by both Western blot and PCR), therefore promoting the generation of S1P. However, the exact involvement of PAR1 during this process remains to be determined. According to the study by Parker et al. thrombin could cause activation of the small GTPase RhoA *in vivo* (47). This is of particular interest, because small GTPases are confirmed to play critical roles in mediating signaling responses of the S1PR (48), and our previous work also demonstrated that RhoA activated by S1PR2-5 dominated the S1P-induced MPO-ANCA-positive IgG-mediated GEnC activation (20). Activation of RhoA signaling induces endothelial barrier disruption by remodeling cytoskeleton and enhancing the formation of contractile stress fibers which are connected to junctions and generate pulling forces within neighboring cells, thus destabilizing cell contact and forming “discontinuous” adherens junctions and tight junctions (49). All these evidences not only suggest a mutual interaction of S1PR-initiated signaling and regulation of S1P synthesis, but also provide clues to the synergistic effect of thrombin and SphK-S1P-S1PR3 signaling on endothelial barrier dysfunction.

Under homeostatic conditions, high levels of S1P in circulation (~1 μM) are a result of its release from endothelial cells and red blood cells, while platelets may only release large amounts of S1P upon platelet activation when endothelial cells are damaged. Thromboxane plays a crucial role in S1P release from human platelets. The coagulation factors thrombin and FXa interact with local S1P availability and its cellular effects at multiple levels (8). A recent study by Campos et al. demonstrated that in rodent models of stroke, the functional S1P receptor antagonist fingolimod could enhance blood-brain barrier integrity and reduce infarct size, indicating S1P as a potential link between coagulation and inflammation system (50). Our previous studies illustrated that the renal expression of S1PRs correlated with both inflammatory and coagulation parameters among AAV patients, and S1P contributed to MPO-ANCA-positive IgG induced GEnC activation through S1PR2-5 and RhoA signaling pathway (18–20). In our current study, we found that thrombin-PAR could interact with SphK-S1P-S1PR signaling pathway to induce GEnC dysfunction in the presence of MPO-ANCA-positive IgG, and S1P could enhance both expression level and activity of TF in MPO-ANCA-positive IgG-treated GEnCs, thus further activating the coagulation system. Therefore, we speculate that in AAV, S1P might act as a mutual link between inflammation and coagulation system. Blockade of this Sphk1-S1P-S1PR3 signaling pathway may be critical for attenuating the pathological processes associated with over-activation of both coagulation system and inflammation system in AAV.

## Conclusions

In conclusion, thrombin is able to enhance MPO-ANCA-positive IgG-mediated GEnC activation *via* Sphk1-S1P-S1PR3 signaling pathway. These findings are helpful to figure out the linking role of S1P between coagulation and inflammation in AAV, thus provide potential clues for intervention strategies.

## Ethics Statement

Informed consent from each participant was obtained. The study was conducted in line with the Declaration of Helsinki and was approved by the ethics committees of Peking University First Hospital.

## Author Contributions

X-JS conducted the experiments, analyzed the data, and drafted the manuscript. M-HZ involved in its design, assisted with interpretation of data, and provided suggestion for revising the manuscript. MC conceived of the study, participated in the revision of the manuscript, and provided final approval of the version of the submitted manuscript. All authors read and approved the manuscript.

### Conflict of Interest Statement

The authors declare that the research was conducted in the absence of any commercial or financial relationships that could be construed as a potential conflict of interest.

## Supplementary Material

Figure S1 Detection of SphK1 expression with Western blot.
