# Supplementary figures and images for: Thrombin Contributes to Anti-myeloperoxidase Antibody Positive IgG-Mediated Glomerular Endothelial Cells Activation Through SphK1-S1P-S1PR3 Signaling
Source: Front Immunol. 2019 Feb 15;10:237. doi: 10.3389/fimmu.2019.00237 (PMC6413724; doi:10.3389/fimmu.2019.00237)

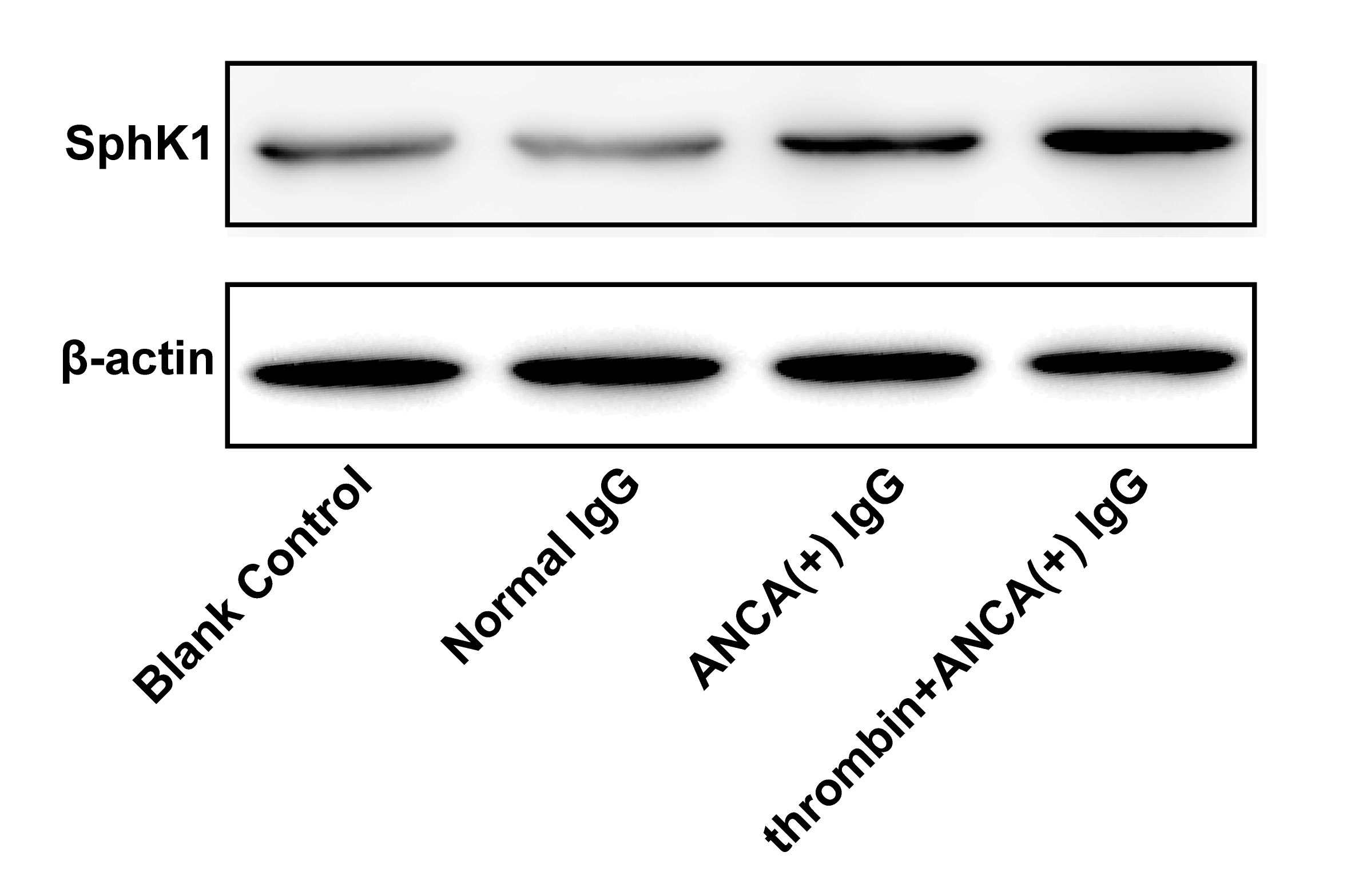

Supplement: Figure S1 — Detection of SphK1 expression with Western blot. [file Image_1.TIF]
